# Identifying resilience promoting factors and sex differences in youth with ADHD across the transition to middle school

**DOI:** 10.1186/s12888-025-07103-9

**Published:** 2025-08-01

**Authors:** Melissa R. Dvorsky, Stephen P. Becker

**Affiliations:** 1 Division of Psychology and Behavioral Health, Children’s National Hospital, 111 Michigan Avenue, Washington, DC 20010 USA; 2 Department of Psychiatry & Behavioral Sciences, And Department of Pediatrics, The George Washington University School of Medicine and Health Sciences, Washington, DC USA; 3 Division of Behavioral Medicine and Clinical Psychology, Cincinnati Children’s Hospital Medical Center, 3333 Burnet Avenue, MLC 10006, Cincinnati, OH 45229 USA; 4 Department of Pediatrics, University of Cincinnati College of Medicine, Cincinnati, OH USA

**Keywords:** Attention-deficit/hyperactivity disorder, Adolescence, Longitudinal, Protective, Social-contextual, Developmental psychopathology, Resilience, Youth, Student

## Abstract

**Background:**

Despite extensive research on risk factors contributing to functional impairment in youth with attention-deficit/hyperactivity disorder (ADHD), there is considerable heterogeneity in outcomes. Some youth experience significant wide-ranging impairments, others experience impairment in specific domains (e.g., academics, social relationships), and still others avoid long-term negative effects. Most existing studies focus on deficits, overlooking strength-based factors that may contribute to positive outcomes for youth with ADHD. A risk-resilience framework offers a valuable approach to identifying promotive and protective mechanisms for youth with ADHD, particularly during the critical developmental transition from elementary school to middle school. Yet research remains limited by cross-sectional methods, small samples, and a failure to consider sex differences in factors supporting well-being.

**Methods:**

This study protocol describes the background and method for a prospective observational study – *Resilience in Student Education (RISE)* – examining individual and social-contextual resilience promoting mechanisms among youth with ADHD from fifth grade through seventh grade, with an added focus on the potentially moderating role of sex in understanding associations between resilience promotive and protective factors for functional outcomes among youth with ADHD.

**Discussion:**

This study also includes formation and engagement of a Youth Advisory Board of adolescents with ADHD (8th-12th grades) to ensure lived experience is incorporated in study measurement, participant engagement/retention, and dissemination of findings with an eye towards how identified promotive and protective factors can be incorporated into interventions to support the well-being of youth with ADHD.

## The need to focus on resilience in youth with ADHD

Historically, developmental/educational science, which has emphasized promotive and protective mechanisms, and the clinical psychology/psychiatry science of attention-deficit/hyperactivity disorder (ADHD), which has primarily emphasized a risk perspective, have developed largely in parallel. These different approaches may explain in part the limitations of current treatments for ADHD which largely focus on alleviating symptoms and associated impairments. Yet there is significant opportunity—and need—to incorporate resilience-promoting factors in our understanding and management of ADHD. By integrating resilience-focused strategies—such as fostering adaptive skills, supportive relationships and environments, and strengths-based interventions—we can shift from merely reducing deficits to helping individuals with ADHD to thrive.

Importantly, resilience-promoting factors are not based solely on the direction in which they are scaled (i.e., not the absence of a problem), but rather promotive and protective factors can be distinguished based on the mechanism of their effect [50] and the extent that they *attenuate* the impact of risk [32]. This requires examining potential promotive or protective factors within the context of the relevant risks [28]. Further, to inform prevention and intervention efforts and maximize impact, it is important for research in this area to prioritize identifying malleable resilience-promoting factors that can be systematically targeted and strengthened within school settings [54, 55].

Decades of research have explored *risk factors* that contribute to educational difficulties in youth with ADHD. Multiple risk factors have been identified, such as executive functioning deficits, oppositional behavior, conflicted parenting, and caregiver psychopathology [4]. This research has led to the development and testing of clinic, school-home, and school-based interventions targeting these very risks, deficits, and impairments [26, 31, 67]. Although these interventions have demonstrated efficacy, sustained effects are lacking. To increase the effectiveness and reach of interventions for youth with ADHD, there is a need to move beyond the “deficit perspective” emphasized in most research which has neglected the evaluation and clinical application of strength-based factors that may contribute to positive outcomes for youth with ADHD.

Although a majority of youth with ADHD experience academic, social, and emotional difficulties, a focused look reveals wide heterogeneity. As a group, youth with ADHD display considerable variation in the severity and onset of academic impairment, peer problems, and the development of co-occurring mental health symptoms [4, 80]. Some youth with ADHD largely avoid these long-term negative outcomes and function well in some domains despite experiencing impairment in other domains [19]. For example, longitudinal studies following youth with ADHD into adulthood have found 20–50% successfully graduate from high school and pursue college [5, 44]. Several studies have examined potential protective factors such as peer acceptance, prosocial activity involvement, and global self-worth that contribute to resilient outcomes for youth with ADHD [18, 29, 30, 41]. This growing literature points to several individual-, social (peer)-, and school-level factors that promote positive academic outcomes for youth with ADHD (see [27], for a review). Unfortunately, the literature base remains small, with most existing studies limited by (1) cross-sectional data, (2) mono-informant designs, (3) modest sample sizes, and (4) predominantly male samples that preclude examining whether certain factors are particularly important for girls or boys with ADHD. As a result, little is known about which factors predict positive academic and socio-emotional outcomes among youth with ADHD and whether sex moderates these relationships.

Risk-resilience research points to developmentally specific risks, promotive factors (i.e., when a factor independently contributes to an outcome by operating in an opposite direction of risk), and protective mechanisms (i.e., a factor that operates by reducing or buffering the effects of risks; interactive effect) [1, 49]. Further, in addition to factors known to promote adjustment for all youth, some factors are especially important for youth with ADHD [27]. For example, the instrumental support from peers (e.g., homework help) that comes with high social acceptance is crucial to accomplishing academically important tasks (e.g., remembering to turn in homework) [16, 29]. However, ADHD is characterized by remarkable heterogeneity, with a failure to understand sex differences – and particularly girls with ADHD – being a key limitation of much ADHD research conducted to date and a top priority for research [76]. Research is needed to identify *malleable promotive and protective factors* for academic and socio-emotional functioning and *how these differ for boys and girls with ADHD*. This is especially important during the middle school transition when youth with ADHD are at increased risk for poor educational outcomes [48].

Accordingly, we are launching a new prospective longitudinal study of children with ADHD, with a focus on promotive and protective factors and intentional recruitment to enroll an approximately 1:1 female-to-male ratio. Participants will be recruited in the fall of fifth grade and followed for two years as they transition from elementary to middle school. As detailed below, a comprehensive multi-informant assessment approach will be used at each timepoint, including ratings of risk, promotive, and protective factors,^1^ and academic and socio-emotional functioning. This study has two overarching objectives:

- Objective 1: Longitudinally evaluate the impact of malleable promotive factors across student, social, and school domains on the academic and socio-emotional adjustment of youth with ADHD across the transition to middle school. This includes examining to what degree promotive factors are associated with *changes* in academic and socio-emotional adjustment to identify mechanisms to be targeted in future assessment, prevention and intervention approaches for ADHD in schools. These models will also consider risk factors (e.g., executive dysfunction, emotion dysregulation) and explore potential interactive effects of protective factors for buffering against risks in predicting adjustment.
- Objective 2: Explore whether sex moderates the impact of promotive and protective processes to identify mechanisms that may be particularly important for girls and boys with ADHD. Explore cascading pathways between individual and social contextual promotive factors in predicting adjustment to identify mechanisms to be targeted in future assessment, prevention and intervention for ADHD in schools. Exploratory aims also include a) examining additional diversity dimensions and individual differences, and b) leveraging student input for incorporating resilience-promoting mechanisms in interventions for youth with ADHD.

## Method

### Overall study design

**Fig. 1:**
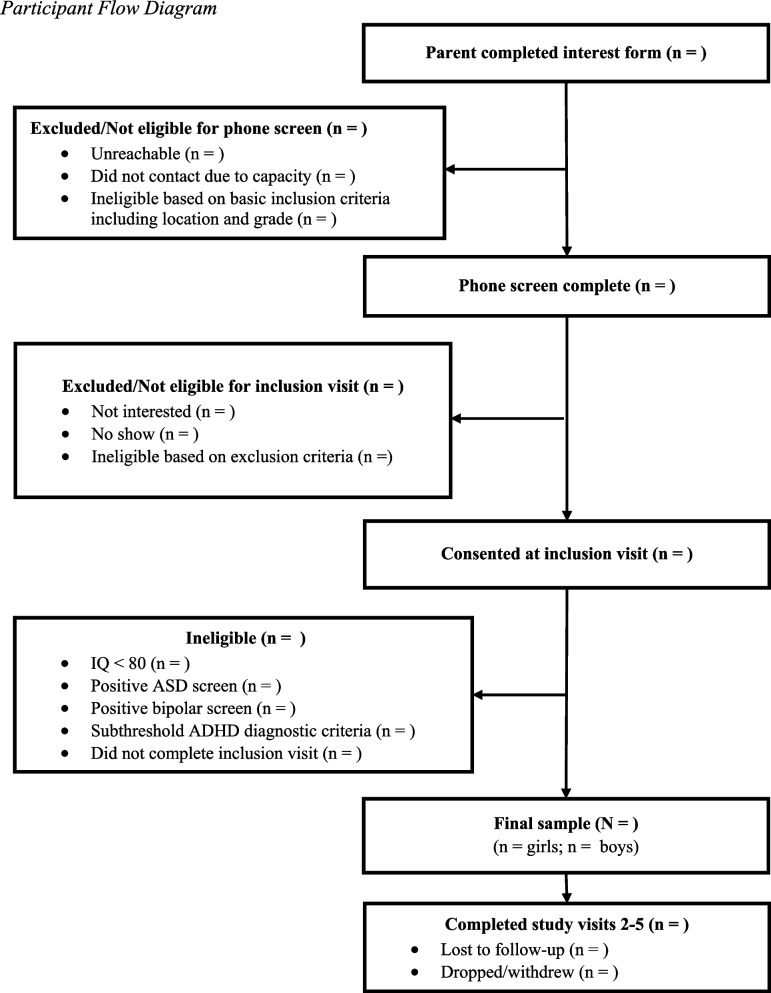
Participant flow diagram

The Resilience in Student Education (RISE) Study is a two-site prospective cohort study of youth with ADHD (see Fig. 1). The study runs from July 2024 through June 2028, encompassing participant recruitment, baseline and follow-up data collection across five timepoints, and primary data analysis. Participants will be recruited through school partnerships and the communities surrounding two sites: Children’s National Hospital (Washington, D.C., U.S.) and Cincinnati Children’s Hospital Medical Center (Cincinnati, Ohio, U.S.). This study is funded by the Institute of Education Sciences (U.S. Department of Education) and approved by the Institutional Review Boards at both institutions. This project underwent peer review as part of the funding process.

### Participants

Participants will include children with ADHD in fifth grade (approximately age 10 years), in addition to their caregivers and teachers. Youth may or may not have been previously identified as having ADHD, and ADHD will be confirmed for all participants prior to enrollment in the study (see below).

### Participant eligibility

#### Inclusion/exclusion criteria

Participants must meet all of the following criteria: (a) consent and assent, (b) full DSM-criteria for ADHD (see next), (c) 5th grade at time of initial enrollment, (d) intelligence quotient (IQ) score ≥ 80, (e) attending core classes in regular education classrooms, and (f) not having a prior diagnosis per caregiver report of autism spectrum disorder, bipolar disorder, a dissociative disorder, or a psychotic disorder or meeting screening criteria for these disorders during the initial eligibility visit.

#### ADHD diagnosis

Youth must meet full DSM-5 criteria [3] for ADHD combined presentation or inattentive presentation as these are the two most common presentations in youth in our sample age range [81] and are more strongly associated than the hyperactive/impulsive presentation with our primary outcome of academic impairment [53]. A participant will be considered to meet diagnostic criteria for ADHD if they meet criteria according to the diagnostic interview conducted with the child’s caregiver which will be the semi-structured Kiddie Schedule for Affective Disorders and Schizophrenia (K-SADS; [42] administered by a trained clinician (e.g., doctoral student, postdoctoral fellows, psychologist under supervision of the principal investigators who are licensed psychologists)). Symptoms identified during the K-SADS may be supplemented by teacher ratings on the Vanderbilt ADHD Diagnostic Teacher Rating Scale (VADTRS; [83]) if (a) at least four ADHD inattentive symptoms are met based on the K-SADS administered to the child’s caregiver, and (b) the teacher endorses at least two four total inattentive symptoms on the VADTRS as occurring “often” or “very often”, including at least two non-overlapping inattentive symptoms not already reported by the caregiver on the K-SADS. To increase representativeness, participants will be allowed to meet diagnostic criteria for common co-occurring mental health disorders (e.g., oppositional defiant disorder, anxiety, depression).

### Recruitment and study timeline

Participants will be recruited in two cohorts. The first cohort will be recruited in September-December 2024, and the second cohort will be recruited in September-December 2025. A total of 300 participants will be recruited, with approximately equal numbers in each cohort and an approximately 1:1 female-to-male ratio. Participants in each cohort will complete five study timepoints, with each timepoint spaced approximately 4–6 months apart. As shown in Table 1, the first and fifth timepoints include in-person visits, and the second through fourth timepoints consist solely of measures collected online (virtually) to increase feasibility for both study staff and participants. Figure 1 provides a flow diagram of recruitment steps and Table 1 provides an overview of study timepoints.

**Table 1:** Study timeline

	Sep – Dec 2024	Mar – June 2025	Sep – Dec 2025	Mar – June 2026	Sep – Dec 2026	Mar – June 2027	Sep – Dec 2027	Mar – June 2028
Cohort 1	Timepoint 1 (5th grade)	Timepoint 2 (5th grade)	Timepoint 3 (6th grade)	Timepoint 4 (6th grade)	Timepoint 5 (7th grade)			
Cohort 2			Timepoint 1 (5th grade)	Timepoint 2 (5th grade)	Timepoint 3 (6th grade)	Timepoint 4 (6th grade)	Timepoint 5 (7th grade)	
Youth Advisory Board (YAB)	Formation	Meeting 1	Meeting 2		Meeting 3	Meeting 4		Meeting 5

Timepoints 1 and 5 include in-person visits; timepoints 2-4 include ratings collected virtually

Recruitment will occur via partnerships with school districts surrounding the two research sites, as well as through advertisements distributed in the community and medical centers where the study is being conducted. Recruitment materials will include descriptions of ADHD behaviors (e.g., inattention, disorganization) so that enrollment focuses on current, observed behaviors, rather than previous diagnoses. School district partners will be encouraged to share information about the study (e.g., study flyers) broadly with caregivers of all fifth grade youth to ensure that all potential eligible fifth-grade youth/families have the opportunity to participate, irrespective of whether or not they have been previously identified as having ADHD and/or are receiving associated school services (e.g., 504 Plan or Individualized Education Program [IEP]). Interested caregivers will first complete an e-screener that includes contact information and broad inclusion/exclusion criteria, potentially eligible families will be contacted by phone so they can learn more about the study and have additional assessment of inclusion/exclusion criteria. During the phone screen, caregivers will be administered the 9-item inattentive subscale of the Vanderbilt ADHD Diagnostic Parent Rating Scale (VADPRS; [83]) to assess the presence of current ADHD inattentive symptoms (if applicable, caregivers will be asked to rate their child’s behavior when not taking medication). If the caregiver endorses four or more symptoms as occurring “often” or “very often” for their child, the child will be considered to potentially meet criteria for ADHD. Eligible families who remain interested will be scheduled for the in-person inclusion/exclusion visit which can be completed at their child’s school, a local library, or the investigator’s clinic. Transportation will be offered to all families. The research team will selectively recruit and enroll to enroll an approximately equal number of females and males. Caregiver consent and child assent will be obtained prior to administration of any study measures, and caregivers will also sign a release of information with permission to gather teacher ratings and school records (e.g., report card grades). For families that are eligible, the inclusion/exclusion visit is the timepoint 1 visit and all measures for the initial timepoint are collected.

Approximately four to six months after timepoint 1 (March—May, second half of fifth grade), caregivers, children, and teachers will complete timepoint 2 measures online. The same informants will also complete measures online for the timepoint 3 (September—December, first half of sixth grade) and timepoint 4 (March—May, second half of sixth grade). Finally, families will complete an in-person visit at timepoint 5 (September—December, first half of seventh grade), and teacher ratings will again be obtained. If families move away from the area where the research is being conducted, they will be invited to complete the fifth timepoint virtually to the extent possible. School records, including report cards, will be gathered at the end of the fifth, sixth, and seventh grade years. Note that in the U.S., the progression from fifth to sixth grade is generally the transition from elementary school to middle school for most school districts.

#### Participant retention

We will use multiple strategies to retain the sample during the proposed study, including (1) adequate participant incentives that increase over time; (2) providing feedback reports to families; (3) acknowledging birthdays and other holidays (e.g., sending birthday and winter holiday cards); (4) offering families the possibility of conducting study visits at the child’s school or a library closer to their home; (5) developing a study website to provide resources and study information for families and school personnel (https://www.studentresilience.org/); (6) study newsletters; (7) providing small toys and/or magnets with study contact information; and (8) collecting contact information of friends and family members who are likely to know the location of participants should they be temporarily out of contact.

## Methodological considerations

### Participant sex

Given sex differences in the impact of both risk and protective factors in previous studies (e.g., [8, 32]), and the relative dearth of studies examining females with ADHD and possible sex differences given historical over-representation of males with ADHD (see above), we will intentionally consider the role of sex in our hypothesized risk-resilience pathways. We will selectively recruit to maintain an approximately 1:1 male-to-female ratio in the proposed study. Recruitment and primary analyses will use sex assigned at birth, and gender identity will also be assessed.

#### Medication

Participants taking psychiatric medication, including psychostimulants used to manage ADHD, will be eligible. Medication is a frequently used treatment among youth with ADHD [59], and so excluding youth taking medication for ADHD would result in a non-representative sample and reduce the generalizability of study findings. In addition, there is significant interest in the degree to which consistency in medication use (and stimulants in particular) during the transition into adolescence adversely or beneficially impacts the functioning of youth with ADHD (see [13, 14]. Thus, we will carefully monitor medication use for all participants throughout the study and will be able to control for medication use in analyses. To facilitate this, at each timepoint caregivers will be asked whether their child has started a new medication or had any changes in their medication treatment.

### Measures

This study uses a multi-method, multi-informant approach, including validated rating scales collected from children, caregivers, and teachers in addition to standardized intelligence and academic achievement testing and the collection of school records. Measures are detailed in Table 2.

**Table 2:** Study measures collected across time-points

**Measures**	**Timepoint**					
	**T1**	**T2**	**T3**	**T4**	**T5**	**Description**
**Student Attitudes and Engagement**						
Behavioral-Emotional Cognitive School Engagement Scale [34]	●	●	●	●	●	A 19-item measure of behavioral, emotional, and cognitive engagement at school
Implicit Theories of Intelligence Scale – Revised [73]	● ◆	● ◆	● ◆	● ◆	● ◆	A 6-item scale assessing an individual’s perception of intelligence as a fluid or concrete concept
Patterns of Adaptive Learning Survey – Revised [56]	● ■	● ■	● ■	● ■	● ■	A 14-item assessment of youth motivation and goal orientations, including performance, avoidance, and mastery approaches
Self-Regulation Strategy Inventory [21]	●	●	●	●	●	A 28-item measure of students’ studying and self-regulation habits
Social-Emotional Health Survey – Secondary [35, 36]	●		●		●	Nine items assessing domains of covitality including self-awareness, persistence, and optimism
Student Subjective Wellbeing Questionnaire [71]	●	●	●	●	●	A 16-item measure of students’ well-being in school including educational purpose, joy of learning, school connectedness, and academic efficacy
Teacher Engagement Report Form [38]	■	■	■	■	■	A 10-item scale measuring youth’s behavioral, cognitive, and affective engagement in the classroom
**Social Support**						
Friendship Quality Questionnaire [2, 75]	●	●	●	●	●	A 23-item rating of supportive friendship qualities, including validation and caring, companionship and recreation, intimate exchange, helping, as well as conflict and betrayal and conflict resolution
Friendship Facilitation Questionnaire [79]	◆	◆	◆	◆	◆	A 20-item scale measuring caregivers’ facilitation of friendships for their child
Multidimensional Scale of Perceived Social Support [15]	● ◆	● ◆	● ◆	● ◆	● ◆	A 12-item rating of social support from family, friends and significant others
School/Community Connectedness						
Community Connectedness Scale [33]	●	●	●	●	●	A 5-item scale assessing youth’s sense of connection in their community
Extracurricular Activity Inventory [58]	◆	◆	◆	◆	◆	A 10-item scale assessing youth’s level of extracurricular involvement, including subscales of frequency, quality, and quantity
Neighborhood Environment Walkability Scale [17]	◆		◆		◆	A 34-item scale rating the satisfaction and environment of neighborhoods
School Climate Measure [87]	●	●	●	●	●	A 42-item scale assessing school connectedness, student–teacher relations, academic support, school social environment, opportunities for student engagement, academic satisfaction, and parent involvement in school
Student–Teacher Relationship Scale [68]	■	■	■	■	■	A 15-item scale assessing teacher’s closeness and conflict with the student
**ADHD Identity and Stigma**						
ADHD Stigma Questionnaire [43]	● ◆				● ◆	A 26-item rating of an individual’s perception of public ADHD-stigmatization
Hyper-Focus Questionnaire	● ◆		● ◆		● ◆	A 3-item form assessing a child’s tendency to hyper-focus
Positive Attitudes Toward ADHD Scale [45]		● ◆		● ◆	● ◆	A 12-item assessment of positive perceptions of some aspects of ADHD
**Academic**						
Academic Competence Evaluation Scales [24, 25]	● ■	● ■	● ■	● ■	● ■	A validated rating scale of academic enabling skills including critical thinking, engagement, motivation, interpersonal skills, and study skills
Classroom Performance Survey [11]	■	■	■	■	■	Two items used from this measure assess the proportion of assignments the student has turned in compared to peers in the classroom
Grades and School Attendance	▲	▲	▲	▲	▲	School grades and attendance collected from school records
Homework Performance Questionnaire [69, 70]	◆ ■	◆ ■	◆ ■	◆ ■	◆ ■	A 27-item scale assessing self-regulation and competence in homework as well as teacher and parent support in homework
Wechsler Individual Achievement Test, Fourth Edition [12]	◄				◄	A standardized assessment of academic skills. The subtests administered are reading comprehension, spelling, word reading, and math fluency
**Socio-Emotional and Mental Health**						
Barkley Deficits in Executive Functioning Scale for Child and Adolescents, Short Form [6]	◆ ■	◆ ■	◆ ■	◆ ■	◆ ■	A 20-item validated measure of youth’s difficulties with executive functions
Behavior Assessment System for Children, Third Edition [72]	● ◆		● ◆		● ◆	A validated assessment of youth’s adaptive and problem behaviors including clinical scales and adaptive skills
Child Behavior Scale [46, 47]	■	■	■	■	■	A 20-item rating of a child’s asocial and prosocial interactions with peers
Emotion Regulation Questionnaire in Children and Adolescents [37]	●		●		●	A 10-item measurement of emotional liability and regulation approaches including cognitive reappraisal and expressive suppression
Emotion Regulation Checklist [74]	◆		◆		◆	A 24-item scale of caregivers’ report of their children’s emotional liability and emotion regulation
Revised Children’s Anxiety and Depression Scale [20]	● ◆	● ◆	● ◆	● ◆	● ◆	A 47-item validated measure of internalizing symptoms (shortened 25-item version at T2-T4) including symptoms of depression, generalized anxiety, separation anxiety, social phobia, panic, and obsessive–compulsive disorder
Vanderbilt ADHD Diagnostic Rating Scale [82–86]	◆ ■	◆ ■	◆ ■	◆ ■	◆ ■	A validated rating scale of youth’s ADHD symptoms, disruptive behavior, internalizing symptoms, and functional impairment
**Youth and Family Background**						
Accountable Health Communities Health-Related Social Needs Screening [9]	◆				◆	A 15-item scale assessing families’ social needs food insecurity, financial strain, and stress; Child Opportunity Index is also calculated from zip code
Pediatric ACEs and Related Life Events Screener [78]	◆				◆	A 17-item scale measuring a child’s exposure to adverse events
Physical Development Scale [40, 66, 77]	●	●	●	●	●	Scale assessing pubertal development
	◆	◆	◆	◆	◆	An assessment of historical and current psychological, psychiatric, and school service usage

● = Child self-report measure; ◆ Caregiver-report measure; ■ Teacher-report measure; ▲School records; ◄Standardized testing; T1 = time 1; T2 = time 2; T3 = time 3; T4 = time 4; T5 = time 5

### Data management and preparation

All data will be collected using secure, web-based platforms (e.g., REDCap) for online surveys and stored on password-protected, encrypted servers. Paper-based assessments (e.g., consent forms, school records) will be digitized and stored in the same secure systems, with original documents kept in locked filing cabinets. Prior to analysis, data will undergo rigorous cleaning and validation procedures, including double data entry and discrepancy checks for any data requiring entry.

### Data analysis

Latent growth curve models will be conducted to examine the type and degree of change in functional outcomes (academic performance, socio-emotional impairment). An exploratory multivariate latent curve model will allow for simultaneously prospective reciprocal relations between constructs representing between-person and within-person effects. Exploratory models will also be conducted using multiple group analyses to examine differences in model parameters for girls and boys. Findings generated from this study will lead to recommendations for how and when schools should assess promotive and protective mechanisms as part of psychoeducational evaluations as well as inform key contextual (i.e., school, social) and individual factors to be enhanced in intervention refinement to maximize the impact on academic outcomes.

Although extensive efforts will be made to minimize attrition and missing data, some attrition is inevitable. Missing data will be handled through use of full-information maximum likelihood estimates and analytic techniques that make use of all available data. Although extensive efforts will be made to minimize attrition and missing data, some attrition is inevitable. We will assess the impact of attrition on sample bias by comparing scores on earlier waves for those who drop out and those who do not. We will incorporate auxiliary variables into models using maximum likelihood estimates to reduce potential bias from missingness even when data are not missing at random [22].

#### Preliminary analyses

The distribution of each variable will be examined to determine the most appropriate analytic model. We anticipate that most variables will be suitable for analyses using maximum likelihood algorithms that are robust to violations of normality (i.e., MLR). Confirmatory factor analyses will be used to determine if multiple measures of related constructs (e.g., friendship quality, peer support, peer acceptance) and measures obtained from different informants (youth, parents, teachers) can be adequately represented by a smaller number of latent variables. We will consider creating composite variables based on multiple measures and sources if they are justified by the results of the factor analyses including tests of measurement invariance over time and across sex. Once the measurement models have been completed, we will conduct analyses to finalize latent curve models to determine the best fitting models of change within and across time (i.e., optimal function of time) for student attitudes/engagement, social support, and school connectedness, academic performance, and socio-emotional functioning. Latent curve models estimate a random intercept and random slope(s) that capture interindividual differences in intraindividual stability and change over time [10, 23]. Selecting the final model will consider model fit, parsimony, and interpretability.

#### Objective 1: investigate reciprocal relations between promotive factors and academic and socio-emotional outcomes

We will incorporate the latent curve models developed for each construct (a) student attitudes and engagement, b) social support, c) school connectedness) into analyses examining reciprocal relations between academic functioning and each promotive mechanisms within the context of a parallel process (multivariate) latent curve model with structured residuals. This extension of the multivariate latent curve models [23] makes it possible to simultaneously examine prospective reciprocal relations between constructs representing both between-person and within-person effects. Between-person effects represent the extent to which initial frequencies of each construct (i.e., academic engagement and grades intercepts) predict changes in the other construct (e.g., engagement and grades slopes). Within-person effects include time-specific relations across constructs. These analyses will enable us to examine the extent to which the degree of a promotive factor (e.g., engagement) at a particular point during the transition to middle school predicts levels of academic performance (e.g., GPA) at the following wave after accounting for between-person differences in trajectories. These effects are modeled through autoregressive models of residuals within the context of the overall growth model. Specifically, we will examine whether the magnitude of the relation between the promotive construct and academic performance changes over time (e.g., the constructs may become increasingly intertwined as youth enter middle school). By removing (freeing) the restriction that the cross-domain regressions are equal over time, this allows them to take on any optimal value supported by the data. We first will estimate a model for both constructs simultaneously, then conduct tests of cross-construct relations at the level of the latent factors and of the time-specific residuals and test equality constraints on the cross-lagged regressions. Finally, we expand the multivariate model to include covariates and risk factors. For each step we conduct likelihood ratio tests to formally evaluate the change in model fit relative to the inclusion of additional parameters or the imposing of parameter constraints [23]. We will interpret findings based on the significance of individual parameters, and effect sizes representing the overall variance accounted for by each variable.

#### Objective 2: explore the extent to which sex moderates patterns of stability and change in promotive factors, socio-emotional and academic functioning, and their reciprocal relations

Building on the primary models in Objective 1, we will use multiple group moderation analyses to test for systematic differences of promotive factors and academic/socio-emotional functioning as a function of sex. Consistent with methods from Bauer and Hussong [7], we will evaluate model constraints on the magnitude of within-person prospective effects across time and by sex. This will test whether the strength of reciprocal relations between the two constructs is similarly constant or systemically weakened or strengthened over time for boys or girls with ADHD.

#### Statistical power

To estimate statistical power, we computed power bands for specific parameters within the proposed latent curve models [61] and for omnibus tests of model fit [51] and all power estimates exceed the accepted values of 0.80. Based on power curves for growth models in Muthén & Curran [61], our proposed sample size (*n* = 300) provides power exceeding 0.80 to detect a small effect size in the mean and variance of slope factors for all our proposed models. We can extend these calculations to compute power for rejecting a misspecified model using omnibus measures of fit. Drawing on MacCallum et al. [51], power exceeds 0.90 for rejecting a truly misspecified model using a test of"close fit"(i.e., RMSEA < 0.05) for the primary analyses (bivariate LCM). Power estimates increase to > 0.95 for the more complex models given the higher associated degrees-of-freedom. Taken together, we predict high power to detect a small effect size both at the level of a given parameter and at the omnibus level of overall model fit. These high levels of power further suggest there is sufficient observed information to support stable model estimation [52].

### Youth Advisory Board (YAB)

In addition to the 300 research participants, we will create a Youth Advisory Board (YAB) composed of 10–15 youth with ADHD (grades 8–12) who will be active partners throughout the study. The investigative team and the YAB will meet virtually at least five times throughout the duration of the research project. Consistent with best practices for participatory research with adolescents [63, 64], we will partner with the YAB to review and discuss assessment, measures, data analysis and interpretation of findings, and implications for interventions supporting youth with ADHD. This student-centered methodology is informed and inspired by the broader neurodiversity perspective recognizing the diverse and multifaceted nature of ADHD [62, 65]. The YAB will empower youth with ADHD as active participants in research and treatment development decisions, promote a focus on well-being, and respect individual differences and cultural contexts to develop more effective and inclusive interventions.

## Discussion

In context of the Methods described above, it is important to note methodology strengths as well as key methodological considerations and limitations.

### Methodological strengths, considerations, and limitations

Strengths of this study include the large sample size involving two sites, intentional recruitment of an approximately equal number of boys and girls with ADHD, and focus on resilience factors and mechanisms that remain far understudied in ADHD-focused research. In addition, this study uses a rigorous multi-method, multi-informant longitudinal design to gather robust empirical data while also engaging a Youth Advisory Board (YAB) to ensure the lived experience of youth with ADHD is incorporated throughout the study.

In designing this study, we carefully considered the benefits and limitations of enrolling only youth with ADHD, without also enrolling a comparison sample of youth with ADHD. Ultimately, we decided that decades of case–control research has allowed for a large, though admittedly far-from-complete, understanding of differences between youth with and without ADHD. Far less research has focused on sex differences in youth with ADHD, and our current understanding of girls with ADHD largely stems from studies with female-only samples (e.g., Berkeley Girls with ADHD Longitudinal Study [BGALS]; [39]), or from samples that were disproportionately male and/or limited to ADHD combined presentation which likely underrepresents females with ADHD (e.g., Multimodal Treatment of ADHD [MTA] Study; [60]). Thus, with the resources available for this study, we decided to prioritize inclusion to only youth with ADHD that would allow for enrolling an approximately equal number of boys and girls with ADHD.

This study excluded some conditions that often receive unique school and other services including youth with bipolar disorder, low cognitive functioning, and autism. Limitations also include the exclusion of non-English speaking families due limited availability of bilingual staff and validated translated measures. In addition, the second through fourth timepoints include a shortened battery of rating scale measures that can be readily completed online (virtually), which maximizes feasibility and reduces burden for families but in doing so also limits the methods collected at these timepoints (e.g., no standardized academic achievement testing). Finally, the study design and funding will follow participants into 7th grade (approximately age 12); although not a limitation per se, the predictive and transactional associations examined in this study will be limited to an important, though somewhat narrow, period of development, though if additional funding is secured this would allow for additional engagement of participants as they transition into adolescence.

## Conclusions

This study will examine whether resilience factors (e.g., school connectedness, social support, student engagement) predict academic and socio-emotional skills for middle school youth with ADHD and whether resilience factors may differ for girls and boys. Although prior research has identified multiple risk factors that increase the probability that youth with ADHD will experience educational difficulties, how strength-based factors may contribute to positive education outcomes is less well understood. A risk–resilience framework is well-suited to examine promotive and protective mechanisms, which are especially important during the transition to middle school, when youth with ADHD are at heightened risk for poor educational outcomes. Additionally, longitudinal designs with large sample sizes of both boys and girls with ADHD can extend knowledge from cross-sectional designs and illuminate differences in outcomes between boys and girls. This study seeks to address the limitations of prior research through a prospective, longitudinal, multi-informant research design with a large sample size and a focus on resilience factors that predict positive academic and socio-emotional outcomes.

## Data Availability

No datasets were generated or analysed during the current study.

## Abbreviations

ADHD: Attention-deficit/hyperactivity disorder
DSM: Diagnostic and Statistical Manual of Mental Disorders
GPA: Grade point average
K-SADS: Kiddie Schedule for Affective Disorders and Schizophrenia
IEP: Individualized Education Program
MLR: Maximum likelihood robust
REDCap: Research Electronic Data Capture
RISE: Resilience in Student Education (RISE) Study
VADPRS: Vanderbilt ADHD Diagnostic Parent Rating Scale
VADTRS: Vanderbilt ADHD Diagnostic Teacher Rating Scale
YAB: Youth Advisory Board

## Footnotes

1 The terms “promotive” and “protective” factors or mechanisms are used to represent individual or contextual processes or resources (e.g., coping strategies, positive teacher-student relationship), which offset the presence of risk. Consistent with past research (e.g., [57]), promotive effects describe main/direct effects, whereas protective effects describe interactive processes (i.e., factors that operate by reducing or buffering the effects of a risk) that reduce the likelihood of a negative outcome (e.g., academic problems). Both promotive and protective mechanisms are the focus of the study described herein because they are strongly related to decreased academic and socio-emotional difficulties in cross-sectional and extant longitudinal research in youth with ADHD [27].
